# A Case Report of Drug Interactions Between Nirmatrelvir/Ritonavir and Tacrolimus in a Patient With Systemic Lupus Erythematosus

**DOI:** 10.7759/cureus.52506

**Published:** 2024-01-18

**Authors:** Nanae Yamamoto, Yuichi Tsuchiya, Mio Fukuda, Hiroaki Niiro, Takeshi Hirota

**Affiliations:** 1 Department of Pharmacology and Therapeutics, Kyushu University Hospital, Fukuoka, JPN; 2 Faculty of Medical Sciences, Department of Medical Education, Kyushu University, Fukuoka, JPN

**Keywords:** cyp3a, cytochrome p450 3a, drug interaction, tacrolimus, ritonavir, systemic lupus erythematosus

## Abstract

Nirmatrelvir/ritonavir is a treatment for COVID-19 consisting of nirmatrelvir, which has anti-SARS-CoV-2 activity, and ritonavir, a booster to maintain blood levels. Ritonavir is known to be a potent inhibitor of cytochrome P450 3A (CYP3A), and interactions with CYP3A-metabolized drugs, such as the immunosuppressant tacrolimus, can be problematic. Ritonavir's inhibition of CYP3A is irreversible due to covalent binding, and its inhibitory effects are expected to persist until replaced by new CYP3A. Here, we report a case where the combination of nirmatrelvir/ritonavir and tacrolimus resulted in toxic tacrolimus blood levels. A patient on tacrolimus for systemic lupus erythematosus (SLE) developed COVID-19 and was prescribed nirmatrelvir/ritonavir. After starting the combination of nirmatrelvir/ritonavir and tacrolimus, the patient's tacrolimus blood levels became abnormally high, leading to the discontinuation of these drugs due to symptoms of tacrolimus toxicity. Even after ritonavir blood levels had fallen below the detection limit, the decline in tacrolimus blood levels was delayed. The CYP3A inhibition of ritonavir persists even when its blood concentration decreases, emphasizing the need for careful consideration of concomitant medications before starting nirmatrelvir/ritonavir therapy. Adjustments or discontinuation may be necessary.

## Introduction

Systemic lupus erythematosus (SLE) is a disease in which the immune system mistakenly attacks healthy tissues, causing inflammation throughout the body. This is caused by the accumulation of immune complexes, such as DNA-anti-DNA antibodies, in the affected tissues. SLE is treated with corticosteroids to correct the abnormalities in the immune system. Patients with SLE who do not respond to corticosteroids, or who experience severe side effects, may be prescribed immunosuppressive drugs [1]. However, it is important to note that these drugs can also weaken the immune system, making patients more susceptible to infections such as COVID-19. Therefore, patients with SLE and those on immunosuppressive therapy are at a higher risk of developing severe COVID-19. To manage the infection, it is recommended that these patients be treated with appropriate COVID-19 drugs.

The newly approved therapeutic drug for COVID-19, called nirmatrelvir/ritonavir (Paxlovid), is made up of two components. The first component, nirmatrelvir, is an inhibitor of the main protease of SARS-CoV-2. The second component, ritonavir, acts as a pharmacokinetic booster to maintain the blood concentration of nirmatrelvir [2]. Nirmatrelvir/ritonavir has a strong affinity for CYP3A, an enzyme that helps to metabolize many different drugs in the body [3]. When nirmatrelvir/ritonavir is co-administered with drugs that are primarily metabolized by CYP3A4, such as tacrolimus, it may inhibit their metabolism, leading to an increase in the blood concentration of the co-administered drugs. Therefore, caution should be exercised when taking nirmatrelvir/ritonavir with other CYP3A-mediated drugs.

Tacrolimus, an immunosuppressive drug, is widely used in the treatment of autoimmune diseases and organ transplantation and has shown excellent therapeutic effects. However, tacrolimus has a narrow effective blood concentration range and is affected by concomitant medications and genetic polymorphisms of metabolic enzymes, making blood concentration monitoring essential for its safe and effective use. Particular attention should be paid to the incidence of adverse effects when blood trough levels exceed 20 ng/mL for prolonged periods [4].

There have been some reports of increased levels of tacrolimus in the blood when taken in combination with nirmatrelvir/ritonavir [5,6]. However, most of these reports have been in patients who have undergone organ transplants, and there is limited information available regarding patients with autoimmune diseases. In addition, there are no reports that mention the blood levels of ritonavir, which is a potent CYP3A inhibitor, and the relationship between ritonavir and tacrolimus levels is unknown. Here, we present a case of a patient with SLE who received a combination of tacrolimus and nirmatrelvir/ritonavir for COVID-19.

## Case presentation

A 43-year-old man who has been taking tacrolimus 3 mg (once daily for eight years) for SLE visited the emergency department with severe headache, nausea, and vomiting. The patient had been diagnosed with COVID-19 four days earlier during a routine visit to a collagen medicine clinic.

He was prescribed nirmatrelvir/ritonavir (300 mg/100 mg) twice daily because his renal function was not impaired. The dose of tacrolimus was reduced from 3 to 2 mg due to possible drug interactions. The patient was not hospitalized and was treated at home. Before his emergency visit to Kyushu University Hospital, he had taken nirmatrelvir/ritonavir four out of six times, two of which resulted in vomiting.

**Figure 1:**
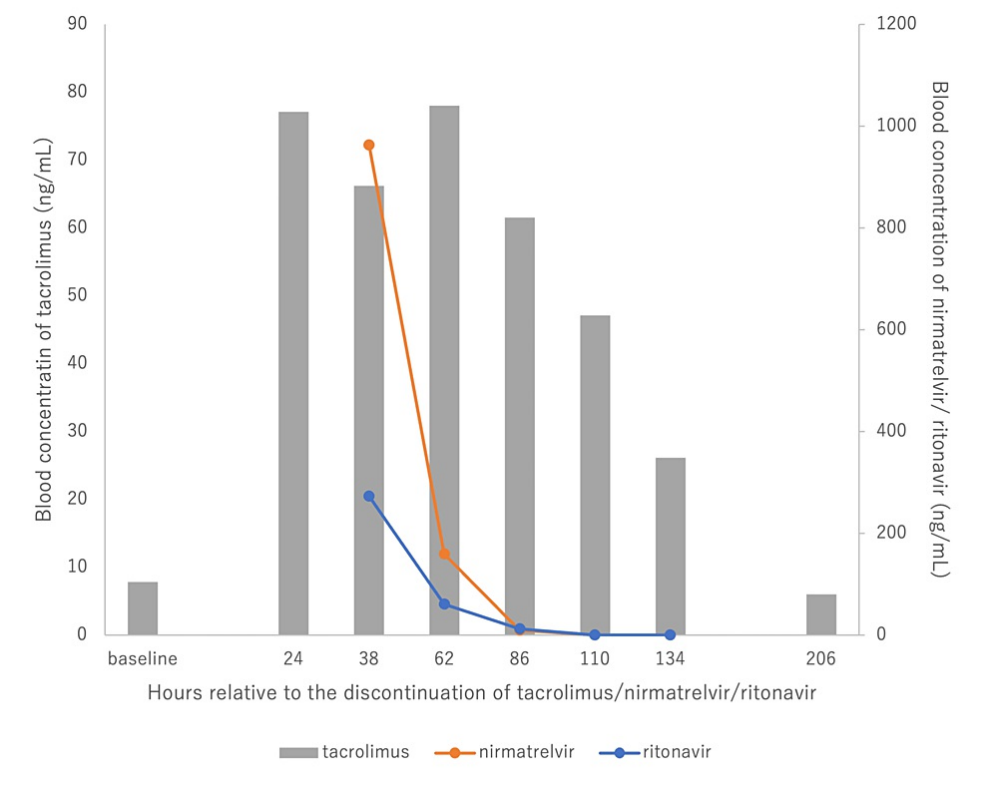
Change in concentrations of tacrolimus, nirmatrelvir, and ritonavir.

Liver and kidney function tests were performed on admission and showed no abnormalities. However, the blood concentration of tacrolimus was found to be 77.1 ng/mL on the day of admission (Figure 1).

**Table 1:** Laboratory data on admission. Alb, albumin; BUN, blood urea nitrogen; Cre, creatinine; AST, aspartate aminotransferase; ALT, alanine aminotransferase; T-Bil, total bilirubin; Mg, magnesium

Lab	Value	Reference range
Alb (g/dL)	4	4.1-5.1
BUN (mg/dL)	10	8.0-20.0
Cre (mg/dL)	1.02	0.65-1.07
AST (U/L)	23	13-30
ALT (U/L)	12	10-42
T-Bil (mg/dL)	0.6	0.4-1.5
Mg (mg/dL)	1.4	1.8-2.7

As a result, both tacrolimus and nirmatrelvir/ritonavir were discontinued on the same day. No additional treatment for COVID-19 was administered during the hospital stay. The patient developed hypomagnesemia, which was treated with magnesium supplementation (Table 1). Until 86 hours after discontinuation of all drugs, nirmatrelvir and ritonavir could be detected in blood, and high tacrolimus levels were maintained. After approximately four days from the discontinuation of the drugs, blood concentrations of ritonavir fell below the detection limit, leading to decreased tacrolimus levels, while the blood concentration of tacrolimus exceeded the toxic range of 20 ng/mL. Even on the ninth day after discontinuation, the blood concentration of tacrolimus had only decreased to the original therapeutic range.

## Discussion

There have been numerous reports of increased tacrolimus blood concentrations with the combination of tacrolimus and nirmatrelvir/ritonavir [5,6]. However, these reports have focused mainly on the measurement of tacrolimus blood concentration. Our report is the first to include the measurement of nirmatrelvir/ritonavir blood concentration. In addition, previous reports have primarily focused on organ transplant patients, whereas our case is the first report on the combination of nirmatrelvir/ritonavir and tacrolimus in a patient with SLE.

We had a patient with SLE whose tacrolimus blood levels rose above the toxic range. This was attributed to the concurrent use of nirmatrelvir/ritonavir with tacrolimus. The patient took tacrolimus once a day with ritonavir for three days. On the second day, the patient vomited after taking the drug, suggesting that the absorption of tacrolimus was insufficient. However, the increase in tacrolimus levels was still above the therapeutic range. This suggests that tacrolimus accumulated almost without being metabolized after taking nirmatrelvir/ritonavir. Tacrolimus can cause headaches, vomiting, and hypomagnesemia [7]. In this case, these symptoms occurred on the second day of taking nirmatrelvir/ritonavir, which was considered to be an adverse effect due to high blood concentrations of tacrolimus.

The package insert for nirmatrelvir/ritonavir advises caution when administering it with tacrolimus due to the inhibitory effect on the metabolic enzyme CYP3A. If drugs such as omeprazole competitively inhibit the metabolism of tacrolimus through CYP3A, the inhibition of metabolism against tacrolimus also resolves as the blood levels of those drugs decrease [8]. On the other hand, ritonavir irreversibly inhibits the enzyme by forming a covalent bond [3], and its inhibitory effect is thought to persist until the ritonavir-bound CYP3A is replaced by a new enzyme.

In this case, the concentration of ritonavir in the blood decreased logarithmically after discontinuation and was below the detection limit (10 ng/mL) after 110 hours. However, the concentration of tacrolimus in the blood remained high, and it took 206 hours after discontinuation of ritonavir to decrease significantly to baseline levels, indicating a substantial delay compared to the decrease in ritonavir blood concentration.

Calculating the half-life of tacrolimus, it was 66 hours (between 62 and 110 hours) and 33 hours (between 110 and 206 hours).

It can be concluded that metabolic inhibition of tacrolimus persisted for 110 hours after discontinuation of ritonavir, given the reported half-life of approximately 35 hours [9]. It was clear that the inhibitory effect of tacrolimus on metabolism persisted in this case.

Recent reports suggest that when nirmatrelvir/ritonavir is taken with immunosuppressive drugs, it may be necessary to stop or reduce tacrolimus on the day nirmatrelvir/ritonavir is initiated [10].

The prescribing information for nirmatrelvir/ritonavir indicates that caution should be exercised when using drugs such as tolvaptan, simvastatin, and apixaban. This is because rapid increases in blood concentrations of these drugs can lead to serious health issues like hypernatremia, rhabdomyolysis, and severe bleeding. Most of these drugs interact via CYP3A [11-13]. Therefore, before starting treatment with nirmatrelvir/ritonavir, it is important to check all precautions for co-administration drugs to ensure safety.

## Conclusions

In this case, it was observed that despite the decrease in ritonavir blood levels, there was a sustained increase in tacrolimus blood levels, presumably mediated by CYP3A inhibition. While caution is often exercised in co-administration due to potential drug interactions, unexpected strong interactions, as seen in this case, may occur. Ritonavir continues to inhibit CYP3A even after its blood levels have decreased. Such drug-metabolizing, enzyme-mediated irreversible interactions deserve special attention. As shown in our case, it is crucial to confirm concomitant medications before starting nirmatrelvir/ritonavir treatment to improve treatment safety. In addition, for drugs such as tacrolimus that are subject to therapeutic drug monitoring (TDM), it is advisable to conduct regular TDM and closely monitor for potential side effects.
